# Design of Oligourea-Based Foldamers with Antibacterial and Antifungal Activities

**DOI:** 10.3390/molecules27051749

**Published:** 2022-03-07

**Authors:** Lorène Tallet, Emilie Frisch, Mégane Bornerie, Claire Medemblik, Benoît Frisch, Philippe Lavalle, Gilles Guichard, Céline Douat, Antoine Kichler

**Affiliations:** 1 Inserm UMR 1121, 11 rue Humann, F-67085 Strasbourg, France; lorene.tallet@gmail.com (L.T.); emilie.frisch@etu.unistra.fr (E.F.); claire.medemblik@inserm.fr (C.M.); philippe.lavalle@inserm.fr (P.L.); 2 Faculté de Chirurgie Dentaire, Université de Strasbourg, 8 rue Sainte Elisabeth, F-67000 Strasbourg, France; 3 CNRS, Bordeaux INP, CBMN, UMR 5248, Institut Européen de Chimie et Biologie, Université de Bordeaux, 2 rue Robert Escarpit, F-33607 Pessac, France; m.bornerie@googlemail.com; 4 CAMB 7199 CNRS, Equipe 3Bio, Faculté de Pharmacie, Université de Strasbourg, F-67401 Illkirch, France; frisch@unistra.fr; 5 Department of Pharmacy and Center for Integrated Protein Science, Ludwig-Maximilians-Universität, Butenandtstrasse 5-13, 81377 München, Germany

**Keywords:** amphiphilic cationic foldamers, oligoureas, oligourea-peptide hybrids, antibacterial activity, antifungal properties

## Abstract

There is an urgent need to develop new therapeutic strategies to fight the emergence of multidrug resistant bacteria. Many antimicrobial peptides (AMPs) have been identified and characterized, but clinical translation has been limited partly due to their structural instability and degradability in physiological environments. The use of unnatural backbones leading to foldamers can generate peptidomimetics with improved properties and conformational stability. We recently reported the successful design of urea-based eukaryotic cell-penetrating foldamers (CPFs). Since cell-penetrating peptides and AMPs generally share many common features, we prepared new sequences derived from CPFs by varying the distribution of histidine- and arginine-type residues at the surface of the oligourea helix, and evaluated their activity on both Gram-positive and Gram-negative bacteria as well as on fungi. In addition, we prepared and tested new amphiphilic block cofoldamers consisting of an oligourea and a peptide segment whereby polar and charged residues are located in the peptide segment and more hydrophobic residues in the oligourea segment. Several foldamer sequences were found to display potent antibacterial activities even in the presence of 50% serum. Importantly, we show that these urea-based foldamers also possess promising antifungal properties.

## 1. Introduction

The emergence of multidrug-resistant bacteria has become an important issue of public health and society. There is therefore an urgent need for new antibacterial compounds effective against bacterial pathogens resistant to current treatments. For example, a challenge in the treatment of *Staphylococcus aureus (S. aureus)* infections is the high prevalence of methicillin-resistant *S. aureus* (MRSA) strains. Different strategies are currently investigated, including seeking new antimicrobial molecules from natural product sources, modification of known antibiotic molecules, and the development of antimicrobial peptides (AMPs). Currently, more than 3000 AMPs have been reported and characterized in the literature [1] including the well-studied magainin and defensin families [2]. These latter peptides are components of the innate immune system of multicellular organisms and are defined as host defense peptides (HDPs). Despite their diversity in sequence and structure, most of the HDPs possess a common structure with an overall positive charge and a substantial number of hydrophobic amino acids conferring them an amphipathic nature. Their length usually varies between 10–50 amino acid residues. The two major abundant families of naturally occurring HDPs preferentially adopt β-sheet (defensins) or α-helix (cathelicidins) secondary structures upon interacting with the bacterial membranes [2]. Although AMPs exhibit a high potential as therapeutic agents, their application in human medicine has been up to now quite limited (several peptides reached phase III in clinical trials and colistin is used in human and veterinary medicine [3,4]; see Dramp Database). Clinical translation of the AMPs has been mainly hampered by their structural instability and poor stability in biological fluids [5,6].

**Figure 1:**
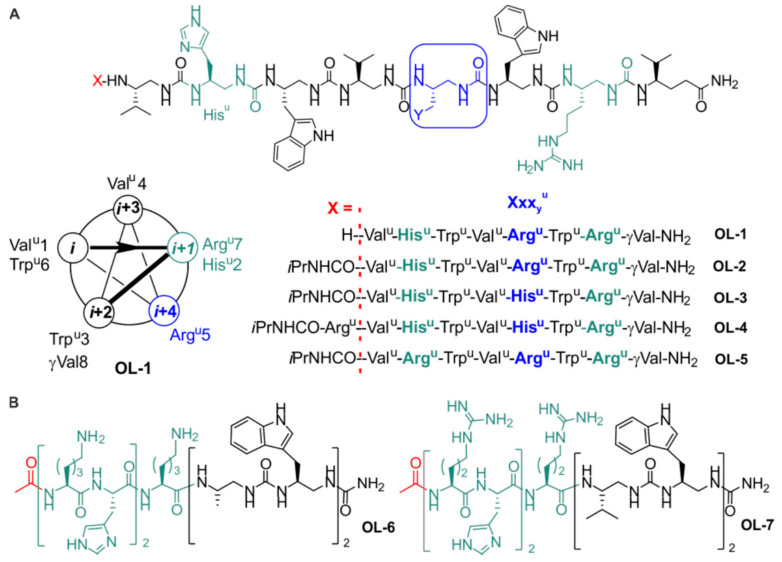
Sequences of the antimicrobial urea-based foldamer candidates. (**A**) Amphiphilic 2.5-helical pure oligoureas evaluated for their antimicrobial activity. A helical wheel representation of oligourea **OL-1** is also shown; *i*Pr stands for isopropyl, superscript “U” indicates urea linkage, and γ is for γ-amino acid. (**B**) Chemical structure of amphiphilic α-peptide–oligourea block cofoldamers **OL-6** and **OL-7**.

To tackle these issues, intense research efforts have been devoted over the last two decades to build synthetic peptidomimetics exhibiting a high propensity to adopt well-defined and predictable folding patterns, referred to as “foldamers” [7]. This research has produced a variety of artificial backbones decorated with biotic side chains and exhibiting an enhanced stability in biological fluids, thus paving the way to the development of innovative medicines [8,9]. In the field of membrane-active foldamers as antimicrobial agents, noteworthy reports include the work on helically folded peptidomimetic backbones such as helical β-peptides [10], α,β-peptide hybrids [11], α-peptoids [12,13], α-peptide/β-peptoid peptidomimetics [14], γ-peptides [15], and sulfono-γ-AA peptides [16]. Concurrently, extended arylamide foldamers have been developed by DeGrado et al. as another family of potent antimicrobial foldamers with remarkable activities and safety profile [17]. This general design was further extended to arylurea foldamers [18]. Compared to other peptidomimetic foldamers, aliphatic urea-based foldamers (of general formula [-CH(R)-CH_2_-NH-CO-NH]*_n_*) [19] (Figure 1) possess several features that make them promising candidates for biological applications as therapeutics: (i) short chain N,N′-linked oligoureas adopt a helical conformation in aqueous solution (2.5-residues per turn) akin to the α-helix [20]; (ii) this conformational preference is largely independent of the primary sequence; (iii) synthetic accessibility allows the incorporation of all proteinogenic amino acid side chains; and (iv) the oligourea backbone is resistant towards enzymatic degradation [21]. 

Because the membrane lytic activity of AMPs is often highly dependent on their secondary structure, the finding that oligoureas display increased biological stability while maintaining their folding propensity in water was found to be promising for the design of AMP mimics. Efforts along this line led to the design of amphiphilic cationic oligoureas as host-defense peptide mimics [22,23,24]. In this first generation antibacterial oligoureas, residues with lysine side chains (i.e., Lys^u^ residues) were used to create a polar cationic surface that roughly covered two-fifth of the helix circumference. Even if arginine and lysine residues possess a positively charged side chain, the incorporation of arginine residues in AMP sequences may lead to more active sequences as a result of the stronger membrane interaction and perturbation properties of guanidinium side chains [25]. This trend is further supported in several publications showing that arginine residues confer higher antibacterial activities to peptides with respect to lysines [26,27]. 

We recently introduced amphiphilic urea-based foldamers containing both arginine and histidine-type residues (Arg^u^ and His^u^) as vectors to deliver plasmid DNA and siRNAs into mammalian cells [28,29,30,31]. In particular, oligourea **OL-3** (Figure 1) and to a greater extent its disulfide-linked dimer (Supplementary Materials Figure S8) were found to efficiently transfect nucleic acids in various cell lines [30]. As CPPs and AMPs share many common chemical and physical features [32], we asked, in this work, whether CPFs (cell-penetrating foldamers) such as **OL-3** and related sequences would also possess antibacterial and antifungal properties. Additionally, we also investigated newly designed amphiphilic peptide–oligourea diblock oligomers which have been shown previously to form regular helical conformations. 

## 2. Results and Discussion

### 2.1. Design and Synthesis of the Foldamers

Starting from oligourea **OL-3** [30], we initially conceived four new sequences presenting different patterns of side-chain distribution along the oligourea helix axis (various Arg^u^/His^u^ ratio), as well as variations in the N-terminal capping mode and main-chain length (Figure 1A). In all amphiphilic oligourea sequences, Arg- and His-type residues (His^u^, Arg^u^) are segregated on one face of the 2.5-helix (Figure 1A). Oligoureas **OL-1** to **OL-5** were synthesized on solid phase using a Boc chemistry strategy as previously described [30] and were obtained in satisfactory yield and high purity (>95%) after RP-HPLC purification (see also Table S1; Figure S1 and Supplementary Materials). To further investigate the interplay between sequence, secondary structure, and antibacterial activity among urea-based foldamers, we decided to investigate hybrid α-peptide–oligourea foldamers which, similar to homo-oligoureas, form well-defined helical structures akin to the α-helix [33]. Hybrid α-peptide–foldamer sequences **OL-6** and **OL-7** (Figure 1B, Figures S2 and S3) were designed to contain each three positive charges (either Lys or Arg residues) and two His residues but without a global amphiphilic nature. In both sequences, polar and cationic residues were concentrated in the α-peptide segment whereas hydrophobic side chains (Ala^u^/Trp^u^ in **OL-6** versus Val^u^/Trp^u^ in **OL-7**) were clustered in the oligourea part at the C-terminus of the peptide segment by analogy to cationic amphiphilic block copolymers [34]. These two hybrid sequences were produced using an azide-type chemistry for the foldamer fragment synthesis combined with a Fmoc-chemistry for the pentapeptide installation on solid support [35]. 

### 2.2. Evaluation of the Antibacterial Activity 

The urea-based foldamers were tested on two Gram-negative bacterial strains, namely *Pseudomonas aeruginosa* (*P. aeruginosa*) and *Escherichia coli* (*E. coli*), and two Gram-positive bacterial strains (*Staphylococcus aureus* and methicillin-resistant *S. aureus* (MRSA)). Of note, *S. aureus*, *P. aeruginosa,* and *E. coli* belong to the ESKAPEE pathogens (*Enterococcus faecium*, *Staphylococcus* *aureus*, *Klebsiella pneumoniae*, *Acinetobacter baumannii, Pseudomonas aeruginosa*, *Enterobacter* spp., *Escherichia coli*), which are responsible for the majority of nosocomial infections and for which new antimicrobial development is urgently needed [36]. 

The results show that all the foldamers **OL-1** to **OL-5** possess minimal inhibitory concentrations (MICs) ≤ 50 μg/mL (Table 1 and Figures S4–S7). Not unexpectedly, the most difficult strain to kill by the oligoureas was the Gram-negative *P. aeruginosa*, whose outer membrane composition allows higher resistance of the bacteria to a wide range of antibiotics [37]. Very interestingly, however, with the exception of **OL-1,** the amphiphilic foldamers killed almost as efficiently (within a factor of two) both strains of *S. aureus* independently whether they were methicillin-resistant or not. 

From these results, several conclusions can be drawn regarding the structure–activity relationship of these molecules: (i) In line with previous results, the N-terminal capping of the 2.5-helix with an isopropyl urea moiety increases the antibacterial activity [24]. Indeed, except on *P. aeruginosa,* **OL-2** performed better than **OL-1**. (ii) Substituting one His^u^ residue in **OL-3** by an Arg^u^ has a positive impact on the antibacterial activity (**OL-2** ≥ **OL-3**). The insertion of one additional Arg-type urea residue at the N-terminus of **OL-3** also increases the activity (**OL-4** ≥ **OL-3**), in agreement with the previous observation. In other words, increasing the overall charge on the surface of 2.5-helix improves the bacterial cell killing capabilities of the resulting foldamers. This trend was further confirmed with the arginine-rich **OL-5** compound which displayed the highest antibacterial responses on the different bacteria lines screened (Table 1). These results are in good agreement with previous findings that have underlined the role of positively charged residues in AMPs for initial electrostatic attraction of the peptides to the negatively charged phospholipid membranes of bacteria [2]. Of note and as shown in Table 2, **OL-5** performed slightly better than its analogue in which Arg^u^ were replaced by Lys^u^ residues (**[Lys^u 2, 5, 7^]OL-5** shown in Figure S8; referred to compound **2** in Claudon et al. [23]) on three out of four strains. This result further supports previous observations showing that Arg residues increase the antibacterial activity with respect to Lys [26,27]. 

**Figure 2:**
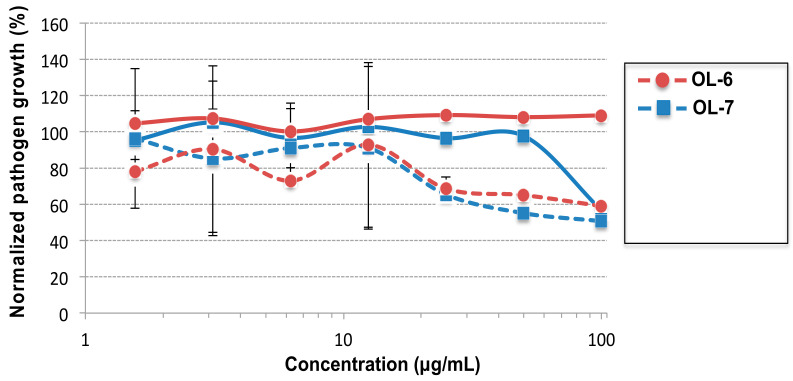
Antibacterial activity of the cofoldamers **OL-6** and **OL-7**. **OL-6** (red lines) and **OL-7** (blue lines) were incubated for 24 h at 37 °C in 100 μL of Mueller–Hinton broth (MHB) medium with either *E. coli* (continuous lines) or *S. aureus* (dashed lines). The control is composed of the bacterial suspension mixed with PBS (=corresponds to 100% growth). Each value corresponds to the mean value of 3 samples.

We next evaluated the antibacterial activity of the amphiphilic α-peptide–oligourea block cofoldamers **OL-6** and **OL-7**. In contrast to **OL-1**/**OL-5**, these two sequences were built with the aim to segregate the positive charges on the peptide while its C-terminus was extended by a hydrophobic foldamer segment for α-helix stabilization [33]. Unexpectedly and despite the same number of guanidinium groups as in **OL-5**, **OL-6** and **OL-7** exhibited no or poor antimicrobial activities on *S. aureus* and *E. coli* with MIC ≥ 100 μg/mL (Figure 2), supporting here the need for a globally amphiphilic helix. 

**Figure 3:**
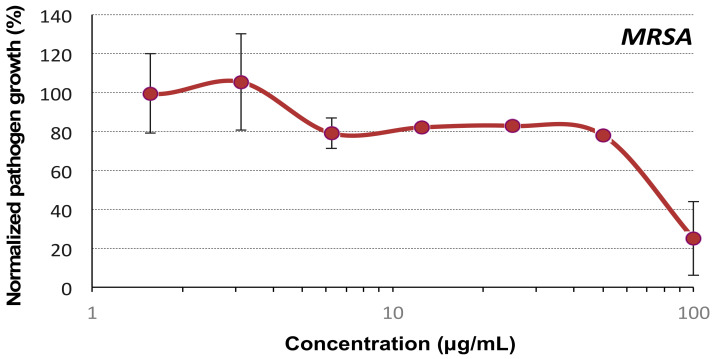
Antibacterial activity of the dimeric urea-based foldamer **DIM-3** on *MRSA*. The foldamer was incubated for 24 h at 37 °C in 100 μL of Mueller–Hinton broth (MHB) medium with the bacterial strain. The control is composed of the bacterial suspension mixed with PBS (=corresponds to 100% growth). Each value corresponds to the mean value of 3 samples.

As mentioned in the introduction, dimerization of **OL-3** via the formation of a bioreducible disulfide bridge strongly enhanced the DNA transfection property of the resulting foldamer [30]. Interestingly, disulfide-dimerization of a magainin analogue also resulted in higher antibacterial activities [38]. We therefore tested the dimeric version of **OL-3** in our assays (**DIM-3**, see Figure S8 for chemical structure). Unexpectedly, this dimer had reduced antimicrobial activity (MIC > 100 μg/mL) (Figure 3) suggesting a different mode of interaction of the **DIM-3** with bacteria compared to **OL-3**. 

This latter result may be explained by the reduced capacity of the dimer to interact with membranes; indeed, recent data obtained by Aisenbrey et al. show that the adsorption of **DIM-3** onto POPC/POPG membranes is reduced at pH 8 compared to **OL-3** monomer [28]. This may suggest that, in aqueous medium at neutral or basic pH, the hydrophobic faces of the two helical segments of **DIM-3** could pack together sufficiently tightly through hydrophobic effect to mask the hydrophobic face of the helix for optimal interaction with the bacterial membrane [28]. Another explanation for the reduced antibacterial activity of **DIM-3** could be the presence of the disulfide bridge itself, in agreement with the recent results of Matile et al., who found that thiol-mediated uptake is inefficient in bacteria and that the addition of thiol reactive groups to antibiotics may actually reduce their activity [39]. 

The cytotoxicity of the four most active oligoureas (**OL-2** to **OL-5**) was assessed using the human cell line MDA-MB-231 by performing an MTS-based cell viability assay. The results showed that exposure of the cells to these foldamers leads to a decrease in the cell viability (Figure S9). The least cytotoxic oligourea was **OL-3** with an IC_50_ of about 50 μg/mL while the IC_50_ for the three other compounds was lower at about 20 μg/mL (Figure S9). In order to determine whether our peptidomimetic foldamers disturb the plasma membrane of the cells, a membrane leakage assay was performed and consisted in monitoring the leakage of the enzyme lactate dehydrogenase (LDH) from the cell cytoplasm into the surrounding culture medium. The results showed a good correlation between the cell viability and the alteration of the membranes (Figure S9) and confirm that **OL-3** is the least cytotoxic foldamer towards mammalian cells.

Together, these results are in good agreement with previous studies that have emphasized that a higher number of arginine residues (i.e., guanidinium moieties) could enhance the antibacterial properties of AMPs but at the expense of a higher cytotoxicity on eukaryotic cells [32].

As **OL-3** proved to exhibit the lowest mammalian membrane permeabilizing activity while displaying good antibacterial activities, we focused on this foldamer for the rest of the study.

### 2.3. Antibacterial Activity in the Presence of Serum

**Figure 4:**
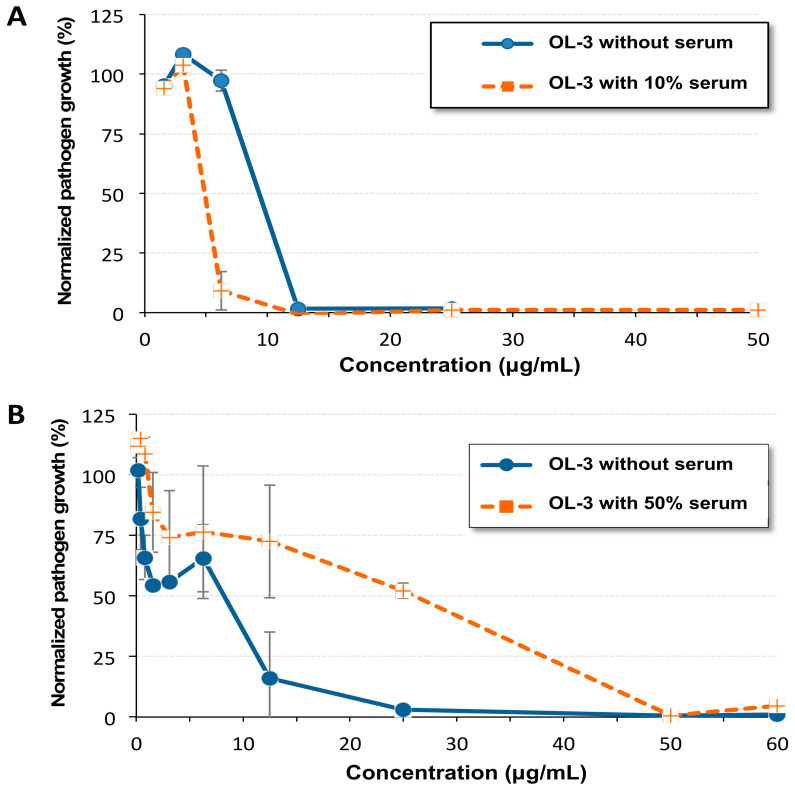
Evaluation of the minimal inhibitory concentration (MIC) of **OL-3** towards *MRSA*. Concentrations are expressed in μg/mL. **OL-3** was incubated for 24 h at 37 °C in 100 μL MHB medium supplemented or not with 10% fetal bovine serum (**A**) or 50% of nondecomplemented fetal calf serum (**B**) with MRSA. Each value corresponds to the mean value of three samples and error bars correspond to standard deviation.

It was recently shown that the activity of different AMPs including peptides such as LL-37 and peptides that are in clinical development is strongly reduced in the presence of 50% human plasma [5]. This is also the case of Pexiganan, a 22-residue antimicrobial peptide developed for topical treatment of infections, which shows a 32-fold loss of activity when tested on *S. aureus* in the presence of 50% bovine serum [40]. To determine whether oligourea **OL-3** is serum sensitive, we first performed an assay using MRSA in the presence of 10% decomplemented fetal calf serum. As reported in Figure 4A, the MIC value of **OL-3** remained unaffected. To further strengthen the experimental conditions, we repeated this assay in the presence of 50% nondecomplemented fetal calf serum. Remarkably, under these harsh conditions, the results showed that the MIC was only reduced by two to four-fold (Figure 4B).

### 2.4. Evaluation of the Antifungal Activity

To the best of our knowledge, urea-based foldamers have never been tested for their antifungal activity and actually only few foldamer backbones (e.g., β-peptides) have been shown to demonstrate antifungal activity [41,42]. For these assays, we chose the two opportunistic human pathogenic fungi *Candida albicans* (*C. albicans*) and *Aspergillus fumigatus.* Both belong to the most common fungal pathogens among immunocompromised patients and they can cause severe systemic infections and are responsible for substantial morbidity and mortality. 

In a first assay, we evaluated the activity of the different foldamers on *C. albicans*. The results reported in the Table 3 show that all the foldamers tested have good activity with a MIC of 12.5 μg/mL or even below (Table 3 and Figure S10). As with bacteria, it is **OL-5** which displayed the highest activity.

**Figure 5:**
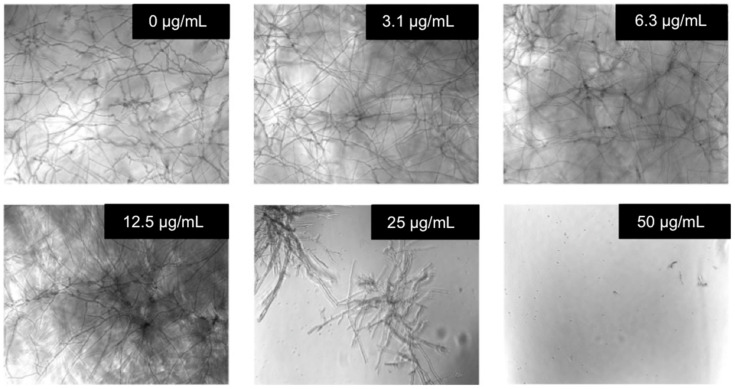
Antifungal activity. The experiment was performed with **OL-3** on *Aspergillus fumigatus* 098. Briefly, spores were resuspended at a concentration of 10^3^ spores/mL in Sabouraud dextrose broth medium. Test samples were incubated with 90 μL of fungal spores (*n* = 3). The suspension was incubated at 30 °C for 24 h without agitation. The fungal growth was then evaluated by microscopy 24 h later.

In order to investigate the extent of activity of **OL-3**, its antifungal property was evaluated on *Aspergillus fumigatus* 098. As shown in Figure 5, the MIC was below 50 μg/mL, a value that is close to the MIC obtained with the commercially available antifungal compound Voriconazole (MIC of 12.5 μg/mL; see Figure S11). Of note, Voriconazole is a broad-spectrum triazole antifungal agent, which is the drug of first choice in the treatment of invasive aspergillosis in adult patients. However, Voriconazole is known to be associated with some adverse effects such as neurotoxicity, visual disturbances, and dermatologic reactions [43].

## 3. Experimental

### 3.1. Synthesis of Amphipathic Foldamer and Related Peptide Hybrids

The foldamers **OL-1–OL-5** were previously reported [23,24,30] and were built on solid support using a Boc chemistry. As an example, **OL-5** was synthesized on a 100 μmol scale starting from MBHA resin. The resin was placed in the reaction vessel (RV), and pre-swollen with DMF for 15–30 min. The first coupling step was performed with a solution of Boc-γ-Val-OH (1.5 eq. relative to resin loading) in DMF (3 mL), with BOP (1.5 eq.) and DIEA (3 eq.), and the resin was shaken for 30 min. This step was repeated once and completion of the coupling was monitored by a Kaiser test. All the following steps were performed under microwave irradiation using a Discover^®^ System (CEM µWaves S.A.S., Orsay, France). The vessel was then placed inside the microwave oven. The temperature was maintained by modulation of power and controlled with a fiber optic sensor. For each following coupling step, a solution of activated Boc-succinimidyl carbamate monomer (3 eq.) and DIEA (6 eq.) in DMF (3 mL) was added to the resin; coupling occurred for 15 min at 70 °C with a power of 25 W. A double coupling was performed systematically. The coupling solution was then filtered off and the resin was washed with DMF (5 × 4 mL). Completion of couplings was monitored with the chloranil test. Boc protecting group was removed using TFA (2 × 5 min). TFA was removed by filtration and the resin was washed with CH_2_Cl_2_ (3 × 4 mL) and with DMF (3 × 4 mL). End capping of the terminal amine with isopropylisocyanate (3 eq.) was performed in DMF (3 mL), in the presence of DIEA (5 eq.) under the same microwave conditions as those used for couplings. Finally, the resin was washed with CH_2_Cl_2_ and dried under nitrogen. Side-chain deprotections and cleavage of the oligomers from the resin were performed simultaneously by treatment with HF (containing 10% p-cresol as a scavenger) for 60 min at 0 °C. The crude oligourea was finally purified by RP-HPLC and pure compound was isolated with a purity above 95% and lyophilized (31 mg, 28%). Compound identity was confirmed by MS analysis (see Supplementary Materials for details).

Amphipathic hybrid peptide–oligourea foldamers **OL-6** and **OL-7** were prepared following an azide-type chemistry for the urea segment and a Fmoc-chemistry for the peptide segment according to a recent literature procedure [35]. As an example, **OL-7** was synthesized on a 50 μmol scale starting from commercially available MBHA-Rink amide resin. The resin was placed in the reaction vessel (RV), and pre-swollen with DMF for 15–30 min. All following steps were performed under inert atmosphere (N_2_) and microwave irradiation on the Discover^®^ system. The four ethylene diamine residues were introduced via their azide-type succinimidyl carbamate activated monomer (1.5 equiv.) dissolved into DMF (2 mL), added to the RV, and immediately followed by DIEA (3 eq.). The RV was then irradiated under microwave (70 °C, 25 W, 15 min). A double coupling was performed systematically. The resin was filtered and washed with DMF (3 × 3 mL). The resin was then filtered off and washed with DMF (3 × 3 mL) and a mixture of 1,4-dioxane/H_2_O (7:3, *v*/*v*, 3 × 3 mL). The Staudinger reduction reaction was performed under microwave irradiation (70 °C, 25 W, 15 min) by swelling the resin in 1,4-dioxane/H_2_O solvent mixture (1.5 mL) and adding 1 M PMe3 solution in THF (10 eq.). The reduction step was systematically performed twice. The resin was then filtered off and washed with DMF (4 × 3 mL). These coupling and reduction steps were monitored with the chloranil test. N-Fmoc-(α)-Xaa-OH (5 eq. relative to the resin loading), DIC (5 eq.), and Oxyma (5 eq.) were next added to RV and the RV heated at 70 °C for Fmoc-Lys(Boc)-OH (70 °C, 25 W, 5 min) or 50 °C for Fmoc-His(Trt)-OH (50 °C, 25 W, 5 min). For coupling the Fmoc-Arg(Pbf)-OH, the reaction was performed at RT and time was extended to 45 min. All couplings were repeated once. The resin was then filtered off and washed with DMF (3 × 3 mL). Fmoc removal was carried out with 20% of piperidine in DMF (2 mL) at RT (1 × 3 then 1 × 7 min). Final acetylation was performed with a mixture acetic anhydride/DCM (1:1, *v*/*v*, 2 mL). The resin was next swelled in a mixture TFA/TIS/H_2_O/(95:2.5:2.5, *v*/*v*/*v*) and let to react for 4 h under slight shaking, then filtered off, and washed with TFA (2 × 2 mL) and CH_2_Cl_2_ (2 × 2 mL). The filtrate was evaporated under reduced pressure and precipitated in cold Et_2_O. The crude oligomer was lyophilized, analyzed on RP-HPLC before to be purified by preparative RP-HPLC to a final purity ≥ 95% and lyophilized (1.3 mg, 2%). Compound identity was confirmed by MS analysis (see Supplementary Materials for details).

### 3.2. Cell Culture

We used the human breast cancer cell line MDA-MB-231. The cells were cultured using RPMI that was supplemented with 100 units/mL penicillin, 100 µg/mL streptomycin, and 10% of fetal calf serum (FCS from HyClone, Fischer Scientific, Illkirch, France). The MDA-MB-231 cells were plated in 48-well plates one day before the experiments were conducted. The cell confluence was around 60% the day of the assay. 

### 3.3. Cell Viability Assay 

The cell viability was determined on MDA-MB-231 cells by performing the MTS assay (3-(4,5-dimethylthiazol-2-yl)-5-(3-carboxymethoxyphenyl)-2-(4-sulfophenyl)-2H-tetrazolium salt) (CellTiter 96^®^ AQueous One Solution Cell Proliferation Assay from Promega). This assay was performed using biological triplicates (same amount of foldamer were added to 3 different wells). Increasing amounts of foldamer diluted in 250 μL/well of RPMI 10% FCS were added to the cells. After 24 h of incubation, the culture medium was removed and replaced with 300 μL/well of RPMI 10% FCS containing the MTS reagent (50 μL/well). After incubation at 37 °C for 1 h and 10 min, 200 μL of medium were withdrawn and used to measure the absorbance at 490 nm (and 680 nm for background). Untreated cells were used as control (100% of cell viability). 

### 3.4. Lactate Dehydrogenase Assay

The membrane permeabilization activity was measured as the leakage of lactate dehydrogenase (LDH) from the cells. Notably, the LDH assay was performed using the same plates as those used for the MTS assay (with *n* = 3). Thereby, foldamers were incubated with MDA-MB-231 cells and after incubation at 37 °C for 1 h and 10 min, an aliquot (50 μL) of the supernatant of each well was used for the measurement of the LDH activity. The experiment was allowed to proceed in order to perform one day later the MTS assay. The LDH release was measured using a commercially available kit (CytoTox-ONE membrane integrity assay from Promega). Untreated cells were used as control. The value of 100% LDH release was obtained by using cells treated with a lysis buffer. Absorbance was measured at 490 nm (and at 680 nm for background). 

### 3.5. Antibacterial Assays

*Staphylococcus aureus* (*S. aureus*, ATCC 25923), *Staphylococcus aureus* S1 Methicillin Resistant (MRSA; this strain was kindly provided by Dr Gilles Prévost, University of Strasbourg), *Escherichia coli* (*E. coli*, ATCC 25922), and *Pseudomonas aeruginosa* (*P. aeruginosa*, ATCC 27853) strains were used to assess the antibacterial properties of the samples. Bacterial strain was cultured aerobically at 37 °C in Mueller–Hinton broth (MHB) medium (Merck, Germany), pH 7.4. One colony was transferred to 10 mL of MHB medium and incubated at 37 °C for 20 h. To obtain bacteria in the mid logarithmic phase of growth, the absorbance at 620 nm of overnight culture was adjusted to 0.001, corresponding to a final density of 5 × 10^5^ CFU/mL. Ninety percent of this culture was incubated with 10% of the test compound at different concentrations. The positive control was composed of 90% bacterial suspension and 10% a mixture of antibiotics (tetracycline: 10 μg/mL and cefotaxime: 0.1 μg/mL in PBS). The negative control was composed of 90% of the bacterial suspension and 10% of PBS, corresponding to our 100% growth. 

In order to test the influence of serum on the minimal inhibitory concentration (MIC), we added 10% decomplemented fetal bovine serum or 50% nondecomplemented fetal bovine serum in MHB medium. After 24 h of incubation at 37 °C with shaking, the optical density at 620 nm of each well was measured. The smallest concentration for which bacterial growth is 0% corresponds to the MIC of the tested compound. 

### 3.6. Antifungal Assays

The *Candida albicans* (*C. albicans*, ATCC 18804) strain was used to assess the antifungal properties of the samples. Briefly, *C. albicans* was cultured aerobically at 30 °C in Sabouraud dextrose broth (SAB) medium (Becton Dickinson, Voisins le Bretonneux, France). One colony was then transferred to 10 mL of SAB medium and incubated at 30 °C for 20 h. The absorbance at 620 nm of overnight culture was adjusted to 0.001 and the tests were performed under the same conditions as those used for bacteria, with the exception of the temperature (30 °C instead of 37 °C) and the culture medium (SAB instead of MHB).

*Aspergillus fumigatus* strain (*A. fumigatus*, ASPFU098 from *Dynamique des interactions hôte pathogène (DIHP)* laboratory—EA 7292, Strasbourg) were tested to evaluate the antifungal properties of foldamers. Spores were resuspended at a concentration of 10^3^ spores/mL in Sabouraud dextrose broth medium. Test samples were incubated with 90 μL of fungal spores. The suspension was incubated at 30 °C for 24 h without agitation. Fungal growth was monitored microscopically after 24 h. Media alone was used as negative control and corresponds to 100% fungal growth; voriconazole (50 μg/mL) was used as a positive control and corresponds to 100% growth inhibition.

## 4. Conclusions

In the present work, we identified new oligourea sequences that possess potent antibacterial activities even in the presence of 50% serum. In addition, we report for the first time that urea-based foldamers such as **OL-3** also possess promising antifungal properties. Finally, our results show that the side-chain composition of the foldamer has to be finely tuned for optimal antimicrobial properties and low cytotoxicity towards mammalian cells.

## Figures and Tables

**Table 1:** Antibacterial activity of the urea-based foldamers (MIC in μg/mL). Each foldamer was incubated for 24 h at 37 °C in 100 μL of Mueller–Hinton broth (MHB) medium with the bacterial strain. The control is composed of the bacterial suspension mixed with PBS (=corresponds to 100% growth). The lowest concentration for which bacterial growth is 0% corresponds to the MIC of the tested compound. Each value corresponds to the mean value of 3 samples. * See Table S2 for MIC in μM.

Oligourea	*S. aureus* ATCC 25923	MRSA	*P. aeruginosa* ATCC 27853	*E. coli* ATCC 25922
**OL-1**	12.5 *	50	12.5	12.5
**OL-2**	6.25	12.5	25	6.25
**OL-3**	12.5	12.5	50	12.5
**OL-4**	6.25	6.25	50	6.25
**OL-5**	12.5	6.25	12.5	6.25

**Table 2:** Antibacterial activity of the urea-based foldamer **OL-5** and its previously reported Lys^u^ counterpart (MIC in μg/mL). Each foldamer was incubated for 24 h at 37 °C in 100 μL of Mueller–Hinton broth (MHB) medium with the bacterial strain. The control is composed of the bacterial suspension mixed with PBS (=corresponds to 100% growth). The lowest concentration for which bacterial growth is 0% corresponds to the MIC of the tested compound. Each value corresponds to the mean value of 3 samples.

Oligourea	*S. aureus* ATCC 25923	MRSA	*P. aeruginosa* ATCC 27853	*E. coli* ATCC 25922
**OL-5**	12.5	6.25	12.5	6.25
**[Lys^u 2, 5, 7^]OL-5**	12.5	12.5	25	12.5

**Table 3:** Antifungal activity of the foldamers (MIC in μg/mL). One colony of *C. albicans* was transferred to 10 mL of Sabouraud dextrose broth (SAB) medium and incubated at 30 °C for 20 h. The absorbance at 620 nm of overnight culture was adjusted to 0.001 and the assay was performed under the same conditions as those used for bacteria, with the exception of the temperature (30 °C instead of 37 °C) and the culture medium (SAB instead of MHB). Each concentration of peptide was tested using *n* = 3.

Oligourea	*C. albicans* 18804
**OL-1**	12.5
**OL-2**	12.5
**OL-3**	12.5
**OL-4**	12.5
**OL-5**	6.25

## Data Availability

Data will be made available upon request.

## Supplementary Materials

The following are available online, Table S1. Molecular weight and net charge of the oligoureas; Table S2. Antibacterial activity of the urea-based foldamers (MIC in μΜ); Figure S1. Structure of **OL-5**, RP-HPLC chromatogram of pure compound (30% to 65% of CH3CN, 0.1%TFA in 20 min) and MS analysis; Figure S2. Structure of **OL-6**, RP-HPLC chromatogram of pure compound (5% to 80% of CH3CN, 0.1%TFA in 10 min) and HRMS analysis; Figure S3. Structure of **OL-7**, RP-HPLC chromatogram of pure compound (5% to 80% of CH3CN, 0.1%TFA in 10 min) and HRMS analysis; Figure S4. Evaluation of Minimal Inhibitory Concentrations (MIC) of five foldamers towards *S. aureus* 25923; Figure S5. Evaluation of Minimal Inhibitory Concentrations (MIC) of five foldamers towards MRSA; Figure S6. Evaluation of Minimal Inhibitory Concentrations (MIC) of five foldamers towards *P. aeruginosa*; Figure S7. Evaluation of Minimal Inhibitory Concentrations (MIC) of five foldamers towards *E. coli;* Figure S8. Structure of **OL-5** analogue [**Lys^u 2, 5, 7^**]**OL-5** and of **DIM-3**, the dimer of **OL-3**; Figure S9. LDH and MTS assays performed on the human cell line MDA-MB-231; Figure S10. Evaluation of Minimal Inhibitory Concentrations (MIC) of five foldamers towards *C. albicans;* Figure S11. Antifungal activity in μg/mL of Voriconazole evaluated on *Aspergillus fumigatus* 098.

## Sample Availability

Please inquire about the quantities available from the authors.

## References

[1] Wang G., Li X., Wang Z. (2016). APD3: The antimicrobial peptide database as a tool for research and education. Nucleic Acids Res..

[2] Yeaman M.R., Yount N.Y. (2003). Mechanisms of antimicrobial peptide action and resistance. Pharmacol. Rev..

[3] Chen C.H., Lu T.K. (2020). Development and Challenges of Antimicrobial Peptides for Therapeutic Applications. Antibiotics.

[4] Mookherjee N., Anderson M.A., Haagsman H.P., Davidson D.J. (2020). Antimicrobial host defence peptides: Functions and clinical potential. Nat. Rev. Drug Discov..

[5] de Breij A., Riool M., Cordfunke R.A., Malanovic N., de Boer L., Koning R.I., Ravensbergen E., Franken M., van der Heijde T., Boekema B.K. (2018). The antimicrobial peptide SAAP-148 combats drug-resistant bacteria and biofilms. Sci. Transl. Med..

[6] Mourtada R., Herce H.D., Yin D.J., Moroco J.A., Wales T.E., Engen J.R., Walensky L.D. (2019). Design of stapled antimicrobial peptides that are stable, nontoxic and kill antibiotic-resistant bacteria in mice. Nat. Biotechnol..

[7] Guichard G., Huc I. (2011). Synthetic foldamers. Chem. Commun. (Camb.).

[8] Gopalakrishnan R., Frolov A.I., Knerr L., Drury W.J., Valeur E. (2016). Therapeutic Potential of Foldamers: From Chemical Biology Tools To Drug Candidates?. J. Med. Chem..

[9] Pasco M., Dolain C., Guichard G., Atwood J.L. (2017). Foldamers in Medicinal Chemistry. Comprehensive Supramolecular Chemistry II.

[10] Hamuro Y., Schneider J.P., DeGrado W.F. (1999). De Novo Design of Antibacterial β-Peptides. J. Am. Chem. Soc..

[11] Schmitt M.A., Weisblum B., Gellman S.H. (2004). Unexpected Relationships between Structure and Function in α,β-Peptides:  Antimicrobial Foldamers with Heterogeneous Backbones. J. Am. Chem. Soc..

[12] Chongsiriwatana N.P., Patch J.A., Czyzewski A.M., Dohm M.T., Ivankin A., Gidalevitz D., Zuckermann R.N., Barron A.E. (2008). Peptoids that mimic the structure, function, and mechanism of helical antimicrobial peptides. Proc. Natl. Acad. Sci. USA.

[13] Shyam R., Charbonnel N., Job A., Blavignac C., Forestier C., Taillefumier C., Faure S. (2018). 1,2,3-Triazolium-Based Cationic Amphipathic Peptoid Oligomers Mimicking Antimicrobial Helical Peptides. ChemMedChem.

[14] Molchanova N., Wang H., Hansen P.R., Høiby N., Nielsen H.M., Franzyk H. (2019). Antimicrobial Activity of α-Peptide/β-Peptoid Lysine-Based Peptidomimetics Against Colistin-Resistant Pseudomonas aeruginosa Isolated From Cystic Fibrosis Patients. Front. Microbiol..

[15] Bonnel C., Legrand B., Simon M., Clavié M., Masnou A., Jumas-Bilak E., Kang Y.K., Licznar-Fajardo P., Maillard L.T., Masurier N. (2020). Tailoring the Physicochemical Properties of Antimicrobial Peptides onto a Thiazole-Based γ-Peptide Foldamer. J. Med. Chem..

[16] Wei L., Wang M., Gao R., Fatirkhorani R., Cai J. (2020). Antibacterial activity of lipo-α/sulfono-γ-AA hybrid peptides. Eur. J. Med. Chem..

[17] Mensa B., Kim Y.H., Choi S., Scott R., Caputo G.A., DeGrado W.F. (2011). Antibacterial Mechanism of Action of Arylamide Foldamers. Antimicrob. Agents Chemother..

[18] Tang H., Doerksen R.J., Tew G.N. (2005). Synthesis of urea oligomers and their antibacterial activity. Chem. Commun. (Camb.).

[19] Yoo S.H., Li B., Dolain C., Pasco M., Guichard G. (2021). Urea based foldamers. Methods Enzymol..

[20] Fischer L., Claudon P., Pendem N., Miclet E., Didierjean C., Ennifar E., Guichard G. (2010). The canonical helix of urea oligomers at atomic resolution: Insights into folding-induced axial organization. Angew. Chem. Int. Ed. Engl..

[21] Teyssières E., Corre J.P., Antunes S., Rougeot C., Dugave C., Jouvion G., Claudon P., Mikaty G., Douat C., Goossens P.L. (2016). Proteolytically Stable Foldamer Mimics of Host-Defense Peptides with Protective Activities in a Murine Model of Bacterial Infection. J. Med. Chem..

[22] Antunes S., Corre J.-P., Mikaty G., Douat C., Goossens P.L., Guichard G. (2017). Effect of replacing main-chain ureas with thiourea and guanidinium surrogates on the bactericidal activity of membrane active oligourea foldamers. Bioorg. Med. Chem..

[23] Claudon P., Violette A., Lamour K., Decossas M., Fournel S., Heurtault B., Godet J., Mély Y., Jamart-Grégoire B., Averlant-Petit M.-C. (2010). Consequences of isostructural main-chain modifications for the design of antimicrobial foldamers: Helical mimics of host-defense peptides based on a heterogeneous amide/urea backbone. Angew. Chem. Int. Ed. Engl..

[24] Violette A., Fournel S., Lamour K., Chaloin O., Frisch B., Briand J.-P., Monteil H., Guichard G. (2006). Mimicking helical antibacterial peptides with nonpeptidic folding oligomers. Chem. Biol..

[25] Mitchell D.J., Kim D.T., Steinman L., Fathman C.G., Rothbard J.B. (2000). Polyarginine enters cells more efficiently than other polycationic homopolymers. J. Pept. Res..

[26] Cutrona K.J., Kaufman B.A., Figueroa D.M., Elmore D.E. (2015). Role of arginine and lysine in the antimicrobial mechanism of histone-derived antimicrobial peptides. FEBS Lett..

[27] Deslouches B., Hasek M.L., Craigo J.K., Steckbeck J.D., Montelaro R.C. (2016). Comparative functional properties of engineered cationic antimicrobial peptides consisting exclusively of tryptophan and either lysine or arginine. J. Med. Microbiol..

[28] Aisenbrey C., Douat C., Kichler A., Guichard G., Bechinger B. (2020). Characterization of the DNA and Membrane Interactions of a Bioreducible Cell-Penetrating Foldamer in its Monomeric and Dimeric Form. J. Phys. Chem. B.

[29] Bornerie M., Brion A., Guichard G., Kichler A., Douat C. (2021). Delivery of siRNA by tailored cell-penetrating urea-based foldamers. Chem. Commun. (Camb.).

[30] Douat C., Aisenbrey C., Antunes S., Decossas M., Lambert O., Bechinger B., Kichler A., Guichard G. (2015). A cell-penetrating foldamer with a bioreducible linkage for intracellular delivery of DNA. Angew. Chem. Int. Ed. Engl..

[31] Douat C., Bornerie M., Antunes S., Guichard G., Kichler A. (2019). Hybrid Cell-Penetrating Foldamer with Superior Intracellular Delivery Properties and Serum Stability. Bioconjug. Chem..

[32] Bahnsen J.S., Franzyk H., Sandberg-Schaal A., Nielsen H.M. (2013). Antimicrobial and cell-penetrating properties of penetratin analogs: Effect of sequence and secondary structure. Biochim. Biophys. Acta.

[33] Fremaux J., Mauran L., Pulka-Ziach K., Kauffmann B., Odaert B., Guichard G. (2015). α-Peptide-Oligourea Chimeras: Stabilization of Short α-Helices by Non-Peptide Helical Foldamers. Angew. Chem. Int. Ed. Engl..

[34] Oda Y., Kanaoka S., Sato T., Aoshima S., Kuroda K. (2011). Block versus Random Amphiphilic Copolymers as Antibacterial Agents. Biomacromolecules.

[35] Cussol L., Mauran-Ambrosino L., Buratto J., Belorusova A., Neuville M., Osz J., Fribourg S., Fremaux J., Dolain C., Goudreau S.R. (2021). Structural Basis for α-Helix Mimicry and Inhibition of Protein-Protein Interactions with Oligourea Foldamers. Angew. Chem. Int. Ed. Engl..

[36] Mulani M.S., Kamble E.E., Kumkar S.N., Tawre M.S., Pardesi K.R. (2019). Emerging Strategies to Combat ESKAPE Pathogens in the Era of Antimicrobial Resistance: A Review. Front. Microbiol..

[37] Breijyeh Z., Jubeh B., Karaman R. (2020). Resistance of Gram-Negative Bacteria to Current Antibacterial Agents and Approaches to Resolve It. Molecules.

[38] Dempsey C.E., Ueno S., Avison M.B. (2003). Enhanced membrane permeabilization and antibacterial activity of a disulfide-dimerized magainin analogue. Biochemistry.

[39] Laurent Q., Berthet M., Cheng Y., Sakai N., Barluenga S., Winssinger N., Matile S. (2020). Probing for Thiol-Mediated Uptake into Bacteria. Chembiochem.

[40] Ge Y., MacDonald D.L., Holroyd K.J., Thornsberry C., Wexler H., Zasloff M. (1999). In Vitro Antibacterial Properties of Pexiganan, an Analog of Magainin. Antimicrob. Agents Chemother..

[41] Karlsson A.J., Pomerantz W.C., Neilsen K.J., Gellman S.H., Palecek S.P. (2009). Effect of sequence and structural properties on 14-helical beta-peptide activity against Candida albicans planktonic cells and biofilms. ACS Chem. Biol..

[42] Karlsson A.J., Pomerantz W.C., Weisblum B., Gellman S.H., Palecek S.P. (2006). Antifungal activity from 14-helical beta-peptides. J. Am. Chem. Soc..

[43] Demir S.O., Atici S., Akkoç G., Yakut N., İkizoğlu N.B., Eralp E.E., Soysal A., Bakir M. (2016). Neurologic Adverse Events Associated with Voriconazole Therapy: Report of Two Pediatric Cases. Case Rep. Infect. Dis..

