# Supplementary material for: Design of Oligourea-Based Foldamers with Antibacterial and Antifungal Activities
Source: Molecules. 2022 Mar 7;27(5):1749. doi: 10.3390/molecules27051749 (PMC8911826; doi:10.3390/molecules27051749)
Supplement: Supplementary file 1 [file molecules-27-01749-s001.zip › molecules-1594478-supplementary.pdf]

SUPPLEMENTARY INFORMATION

Design of oligourea-based foldamers with antibacterial and antifungal activities

Lorène Tallet[1,2], Emilie Frisch[1,2], Mégane Bornerie[3], Claire Medemblik[1,2], Benoît Frisch[4], Philippe Lavalle[1,2], Gilles Guichard[3,*], Céline Douat[3,5,*] and Antoine Kichler[4,*]

1 Inserm UMR 1121, 11 rue Humann, F-67085 Strasbourg, France; lorene.tallet@gmail.com (L.T.); emilie.frisch@etu.unistra.fr (E.F.); claire.medemblik@inserm.fr (C.M.); philippe.lavalle@inserm.fr (P.L.)
2 Université de Strasbourg, Faculté de Chirurgie Dentaire, 8 rue Sainte Elisabeth, F-67000 Strasbourg, France;
3 Université de Bordeaux, CNRS, Bordeaux INP, CBMN, UMR 5248, Institut Européen de Chimie et Biologie, 2 rue Robert Escarpit, F-33607 Pessac, France; m.bornerie@googlemail.com
4 CAMB 7199 CNRS-Université de Strasbourg, Equipe 3Bio, Faculté de Pharmacie, F-67401 Illkirch, France; frisch@unistra.fr
5 Department of Pharmacy and Center for Integrated Protein Science, Ludwig-Maximilians-Universität Butenandtstrasse 5–13, 81377 München, Germany

* Correspondence: g.guichard@iecb.u-bordeaux.fr (G.G.); celine.douat@cup.lmu.de (C.D.); kichler@unistra.fr (A.K.)

Table of Content

1. **Supporting Tables and Figures 3**

Table S1. *Molecular weight and net charge of the oligoureas.* 3

Table S2. *Antibacterial activity of the urea-based foldamers (MIC in μM).* 3

Figure S1: *Structure of OL-5, RP-HPLC chromatogram of pure compound (30% to 65% of CH_3CN, 0.1%TFA in 20 min) and MS analysis.* 4

Figure S2. *Structure of OL-6, RP-HPLC chromatogram of pure compound (5% to 80% of CH_3CN, 0.1%TFA in 10 min) and HRMS analysis.* 5

Figure S3*. Structure of OL-7, RP-HPLC chromatogram of pure compound(5% to 80% of CH_3CN, 0.1%TFA in 10 min) and HRMS analysis.* 6

Figure S4. *Evaluation of Minimal Inhibitory Concentrations (MIC) of five foldamers towards S. aureus 25923. The concentrations are expressed in $\mu g/mL$. Each foldamer was incubated for 24h at 37°C in 100 μL of MHB medium with the bacterial strain. Each value corresponds to the mean value of 3 samples and error bars correspond to standard deviation.* 7

Figure S5. *Evaluation of Minimal Inhibitory Concentrations (MIC) of five foldamers towards MRSA. The concentrations are expressed in $\mu g/mL$. Each foldamer was incubated for 24h at 37°C in 100 μL of MHB medium with the bacterial strain. Each value corresponds to the mean value of 3 samples and error bars correspond to standard deviation.* 7

Figure S6. *Evaluation of Minimal Inhibitory Concentrations (MIC) of five foldamers towards P. aeruginosa. The concentrations are expressed in $\mu g/mL$. Each foldamer was incubated for 24h at 37°C in 100 μL of MHB medium with the bacterial strain. Each value corresponds to the mean value of 3 samples and error bars correspond to standard deviation.* 8

Figure S7. *Evaluation of Minimal Inhibitory Concentrations (MIC) of five foldamers towards E. coli. The concentrations are expressed in $\mu g/mL$. Each foldamer was incubated for 24h at 37°C in 100 μL of MHB medium with the bacterial strain. Each value corresponds to the mean value of 3 samples and error bars correspond to standard deviation.* 8

Figure S8. *Structure of OL-5 analogue [$Lys^{u\ 2,5,7}$]OL-5 and of DIM-3, the dimer of OL-3.* 9

Figure S9. *LDH and MTS assays performed on the human cell line MDA-MB-231. The experiment was conducted as indicated in materials and methods. For the MTS assay, untreated cells were used as control (= 100% of cell viability); for the LDH experiment, untreated cells were used as control. The value of 100% LDH release was obtained by using cells treated with a lysis buffer.* 9

Figure S10*. Evaluation of Minimal Inhibitory Concentrations (MIC) of five foldamers towards C. albicans. The concentrations are expressed in $\mu g/mL$. The foldamer was incubated for 24h at 30°C in 100 μL of Sabouraud Dextrose Broth medium. Each value corresponds to the mean value of 3 samples and error bars correspond to standard deviation.* 10

Figure S11. *Antifungal activity in $\mu g/mL$ of Voriconazole evaluated on Aspergillus fumigatus 098. Briefly, spores were resuspended at a concentration of 10^4 spores/mL in Sabouraud Dextrose Broth medium. Test samples were incubated with 90 μL of fungal spores. The suspension was incubated at 30°C for 24h without agitation. The fungal growth was then evaluated by microscopy 24h.* 10

2. **Materials 11** 11

1. Supporting Tables and Figures

Table S1. Molecular weight and net charge of the oligoureas.

Oligourea	MW	Net charge at pH 7.4
**OL-1**	1367.69	+3
**OL-2**	1452.92	+2
**OL-3**	1433.75	+1
**OL-4**	1618.98	+2
**OL-5**	1470.84	+3
**OL-6**	1348.60	+3
**OL-7**	1488.75	+3

Table S2. Antibacterial activity of the urea-based foldamers (MIC in µM).

Oligourea	***S. aureus* 25923**	***MRSA***	***P. aeruginosa* 27853**	***E. coli* 25922**
**OL-1**	9.14	36.56	9.14	9.14
**OL-2**	4.3	8.6	17.2	4.3
**OL-3**	8.7	8.7	34.8	8.7
**OL-4**	3.86	3.86	30.88	3.86
**OL-5**	8.5	4.25	8.5	4.25

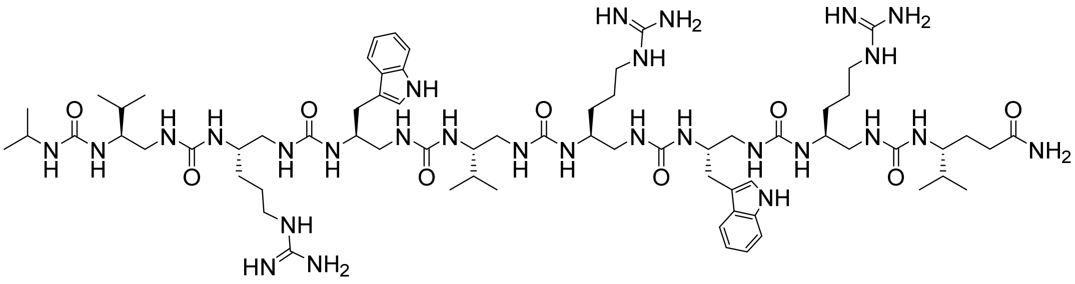

OL-5. Yield after purification 31 mg, 28%; white powder; ESI-MS m/z: $[M+2H]^{2+}$ $C_{68}H_{118}N_{28}O_9$ calcd for: 736.4, found 736.6.

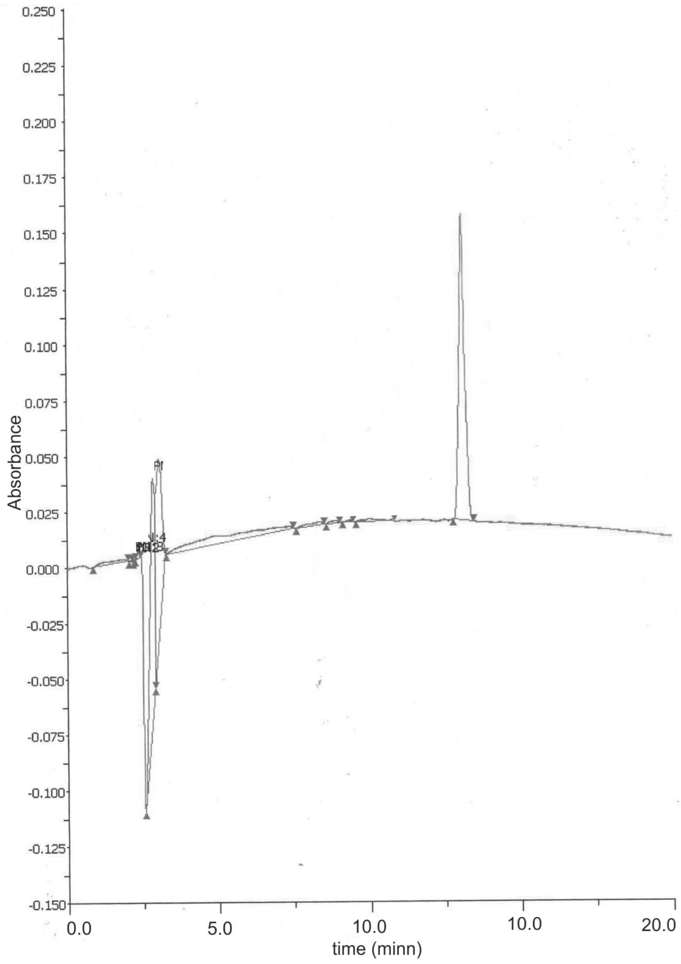

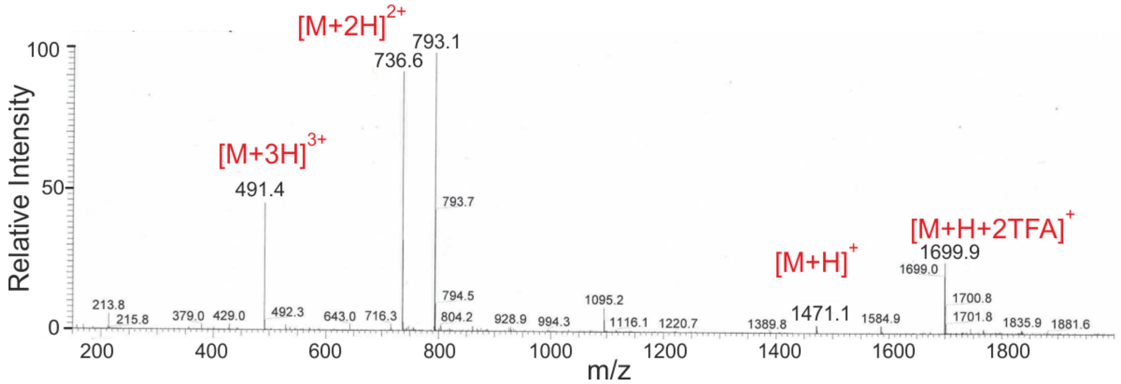

Figure S1. Structure of **OL-5**, RP-HPLC chromatogram of pure compound (30% to 65% of CH_3CN, 0.1%TFA in 20 min) and MS analysis.

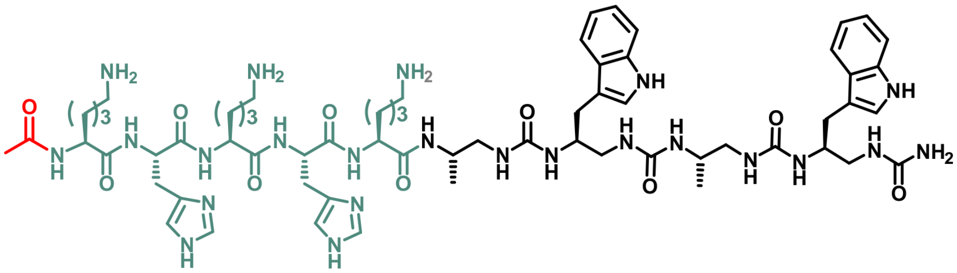

OL-6. Yield after purification 1.3 mg, 2%; white powder; ESI-MS m/z: $[M+2H]^{2+}$ $C_{64}H_{97}N_{23}O_{10}$ calcd for: 674.8049, found 674.8992.

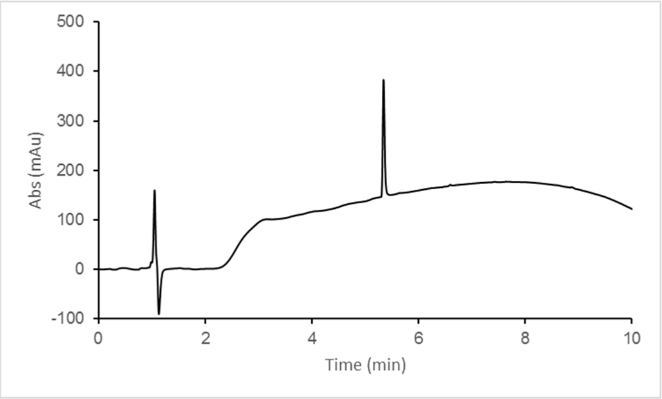

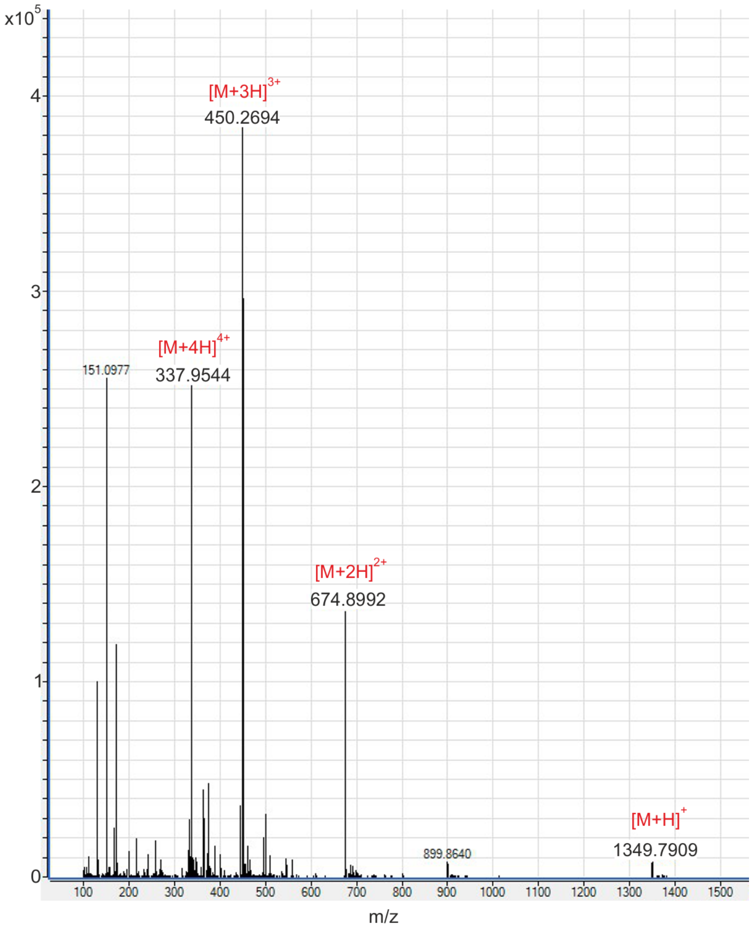

Figure S2. Structure of **OL-6**, RP-HPLC chromatogram of pure compound (5% to 80% of CH_3CN, 0.1%TFA in 10 min) and HRMS analysis.

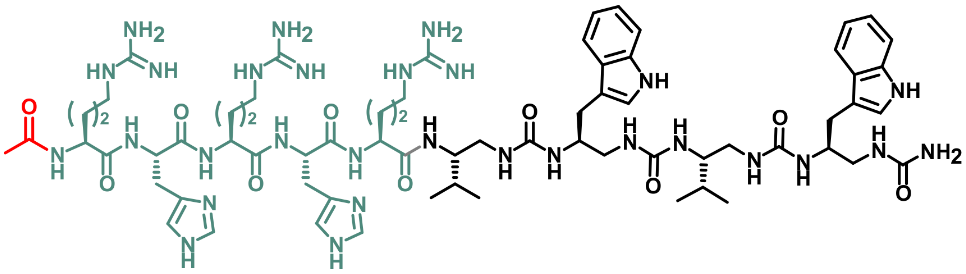

OL-7. Yield after purification 2.28 mg, 3%; white powder; LC-MS *m/z*: $[M+2H]^{2+}$ $C_{68}H_{105}N_{29}O_{10}$ calcd for: 744.8784, found for 744.8749.

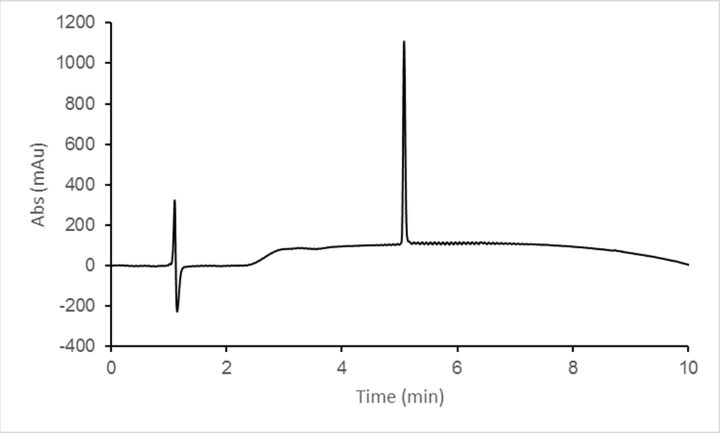

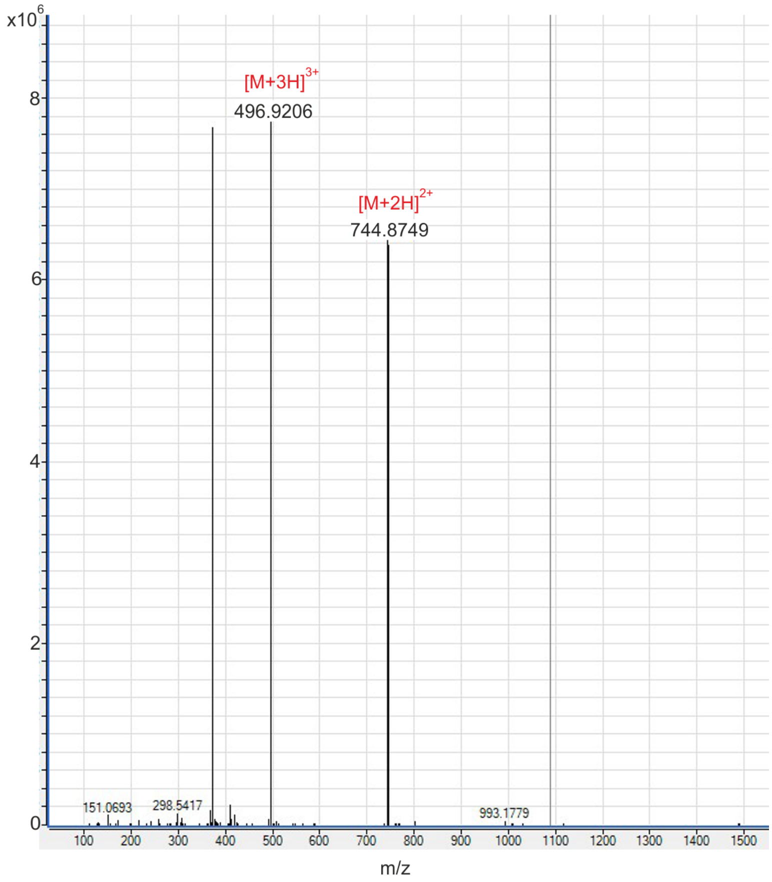

Figure S3. Structure of **OL-7**, RP-HPLC chromatogram of pure compound (5% to 80% of CH_3CN, 0.1%TFA in 10 min) and HRMS analysis.

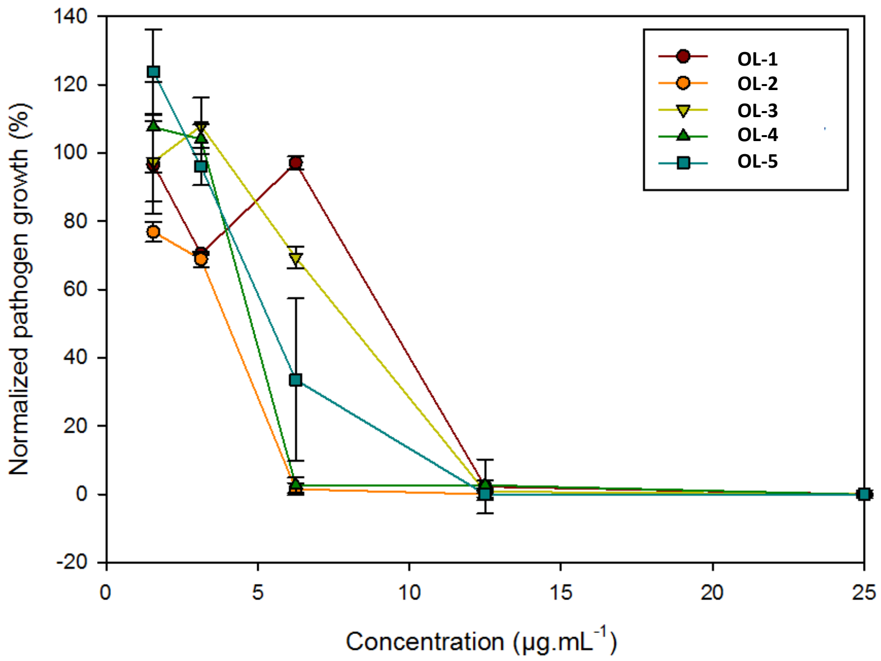

Figure S4. Evaluation of Minimal Inhibitory Concentrations (MIC) of five foldamers towards *S. aureus 25923*. The concentrations are expressed in µg/mL. Each foldamer was incubated for 24h at 37°C in 100 µL of MHB medium with the bacterial strain. Each value corresponds to the mean value of 3 samples and error bars correspond to standard deviation.

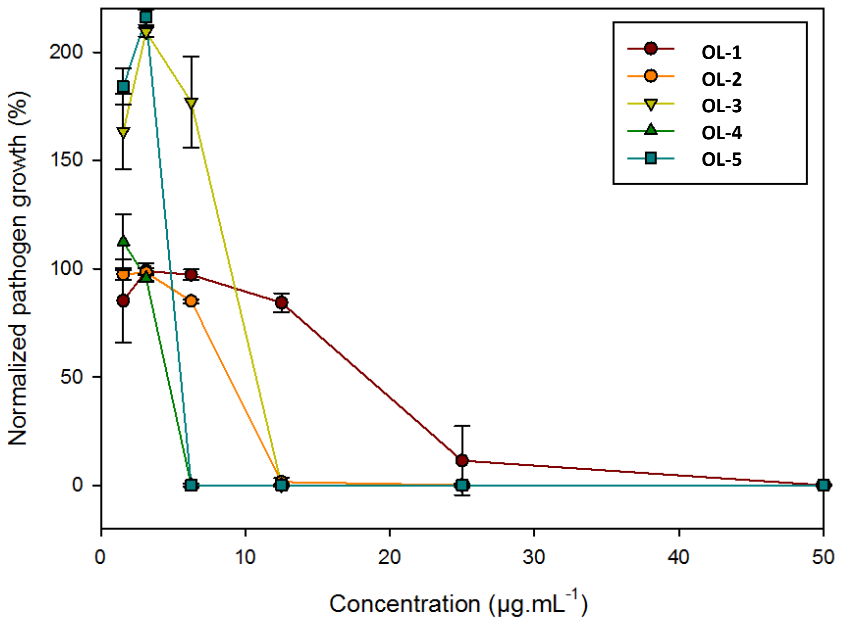

Figure S5. Evaluation of Minimal Inhibitory Concentrations (MIC) of five foldamers towards *MRSA*. The concentrations are expressed in µg/mL. Each foldamer was incubated for 24h at 37°C in 100 µL of MHB medium with the bacterial strain. Each value corresponds to the mean value of 3 samples and error bars correspond to standard deviation.

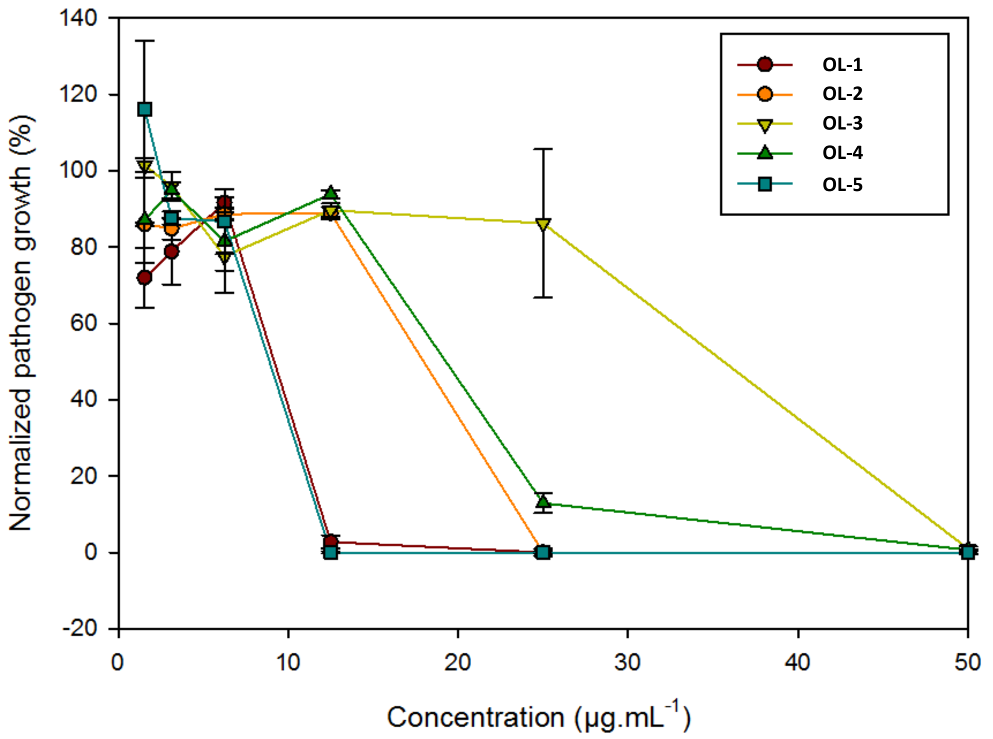

Figure S6. Evaluation of Minimal Inhibitory Concentrations (MIC) of five foldamers towards *P. aeruginosa*. The concentrations are expressed in µg/mL. Each foldamer was incubated for 24h at 37°C in 100 µL of MHB medium with the bacterial strain. Each value corresponds to the mean value of 3 samples and error bars correspond to standard deviation.

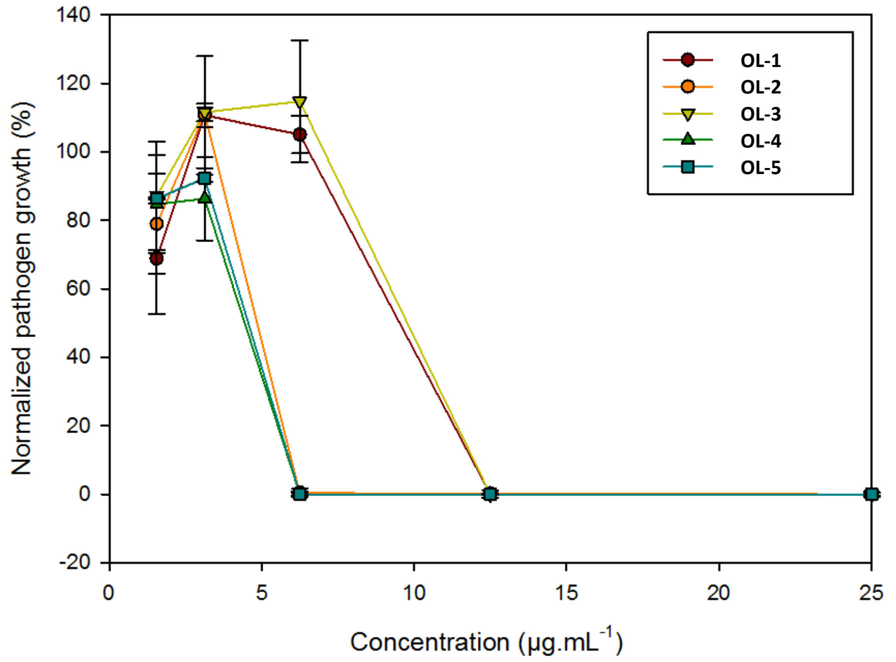

Figure S7. Evaluation of Minimal Inhibitory Concentrations (MIC) of five foldamers towards *E. coli.* The concentrations are expressed in µg/mL. Each foldamer was incubated for 24h at 37°C in 100 L of MHB medium with the bacterial strain. Each value corresponds to the mean value of 3 samples and error bars correspond to standard deviation.

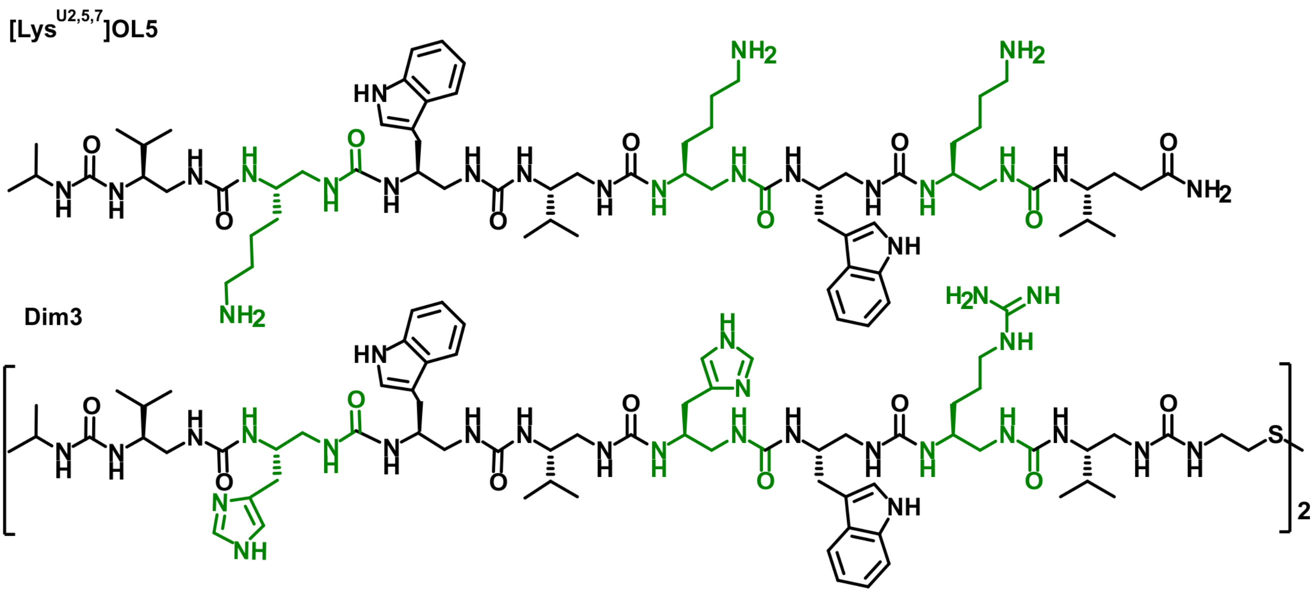

Figure S8. Structure of **OL-5** analogue **[Lys$^{u\ 2,5,7}$]OL-5** and of **DIM-3**, the dimer of **OL-3**.

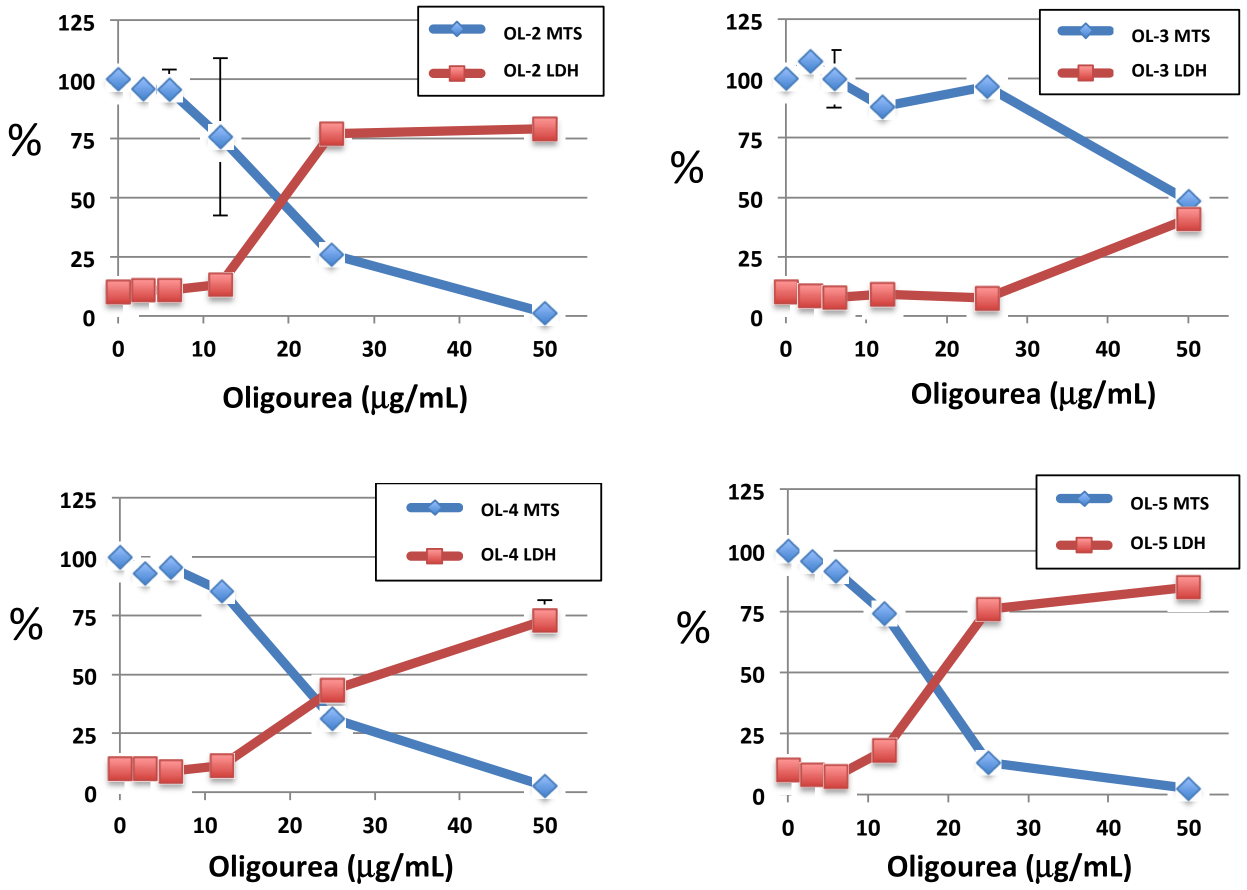

Figure S9. LDH and MTS assays performed on the human cell line MDA-MB-231. The experiment was conducted as indicated in materials and methods. For the MTS assay, untreated cells were used as control (= 100% of cell viability); for the LDH experiment, untreated cells were used as control. The value of 100% LDH release was obtained by using cells treated with a lysis buffer.

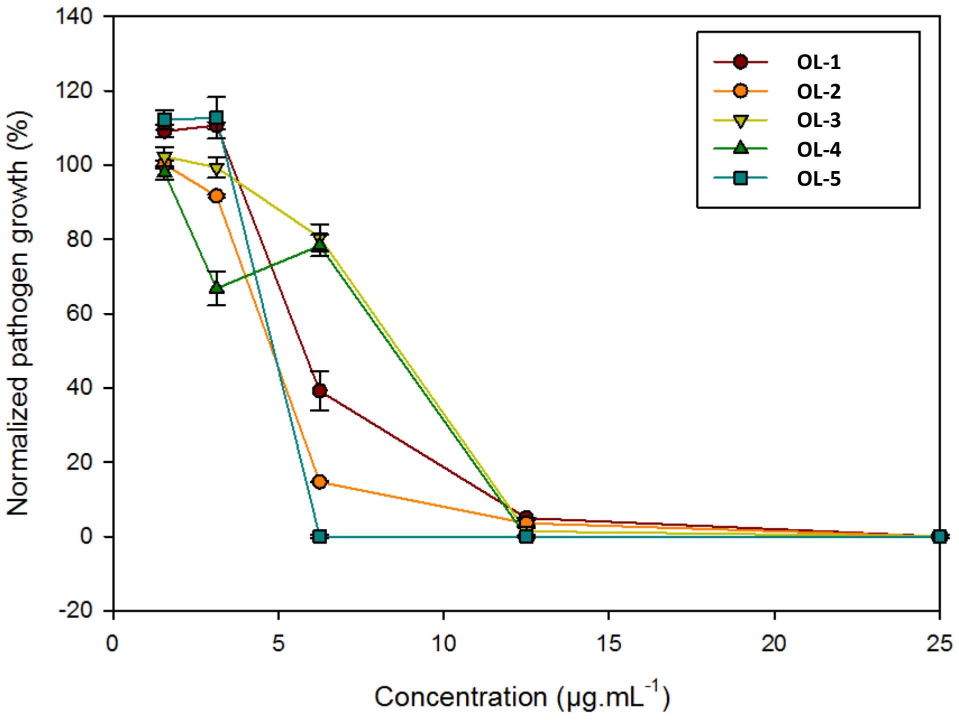

Figure S10. Evaluation of Minimal Inhibitory Concentrations (MIC) of five foldamers towards *C. albicans*. The concentrations are expressed in µg/mL. The foldamer was incubated for 24h at 30°C in 100 µL of Sabouraud Dextrose Broth medium. Each value corresponds to the mean value of 3 samples and error bars correspond to standard deviation.

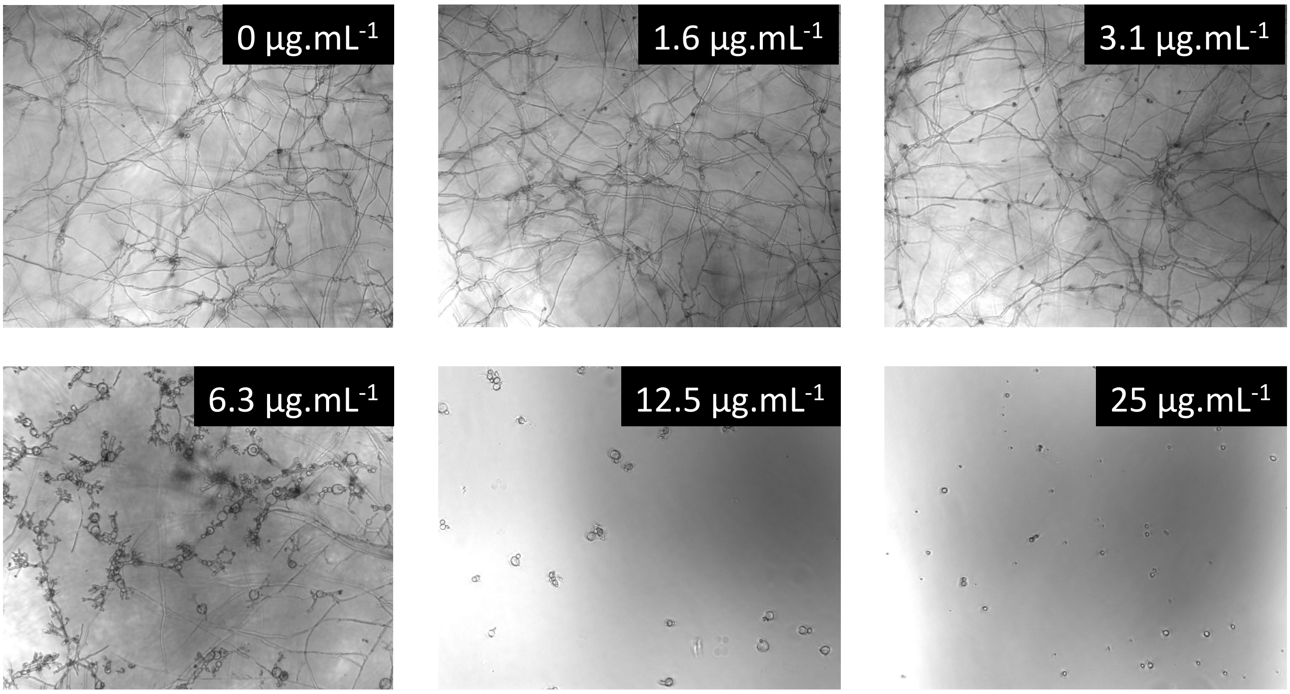

Figure S11. Antifungal activity in µg/mL of Voriconazole evaluated on *Aspergillus fumigatus 098*. Briefly, spores were resuspended at a concentration of 10^4 spores/mL in Sabouraud Dextrose Broth medium. Test samples were incubated with 90 µL of fungal spores. The suspension was incubated at 30°C for 24h without agitation. The fungal growth was then evaluated by microscopy 24h.

2. Materials

Commercially available reagents were used throughout without purification. MBHA-Rink amide and MBHA resins were purchased from Merck Millipore. N,N'-diisopropylethylamine (DIEA) was purchased from Sigma-Aldrich. N-Fmoc amino acids, N,N'-Diisopropylcarbodiimide (DIC) and Ethyl cyano(hydroxyimino)acetate (Oxyma) were purchased from IRIS Biotech GMBH. Benzotriazole-1-yl-oxy-tris-(dimethylamino)-phosphonium hexa-fluorophosphate (BOP) reagent was purchased from PolyPeptide Laboratories France. Solid phase synthesis (SPS) grade organic solvents (DMF, DCM) were used for solid phase synthesis and were purchased from Carlo Erba. Dioxane, RP-HPLC-quality acetonitrile (CH_3CN) were purchased from Sigma Aldrich. MilliQ water was used for RP-HPLC analyses and semi-preparative purifications. Most of the activated succinimidyl carbamate building blocks (N_3 and N-Fmoc protected) used during SPS were prepared using a previously reported procedure. The synthesis of hybrid sequences was performed manually under microwave irradiation on a Discover ® System from CEM (CEM µWaves S.A.S., Orsay, France). RP-HPLC analyses were performed on a Dionex U3000SD using a Macherey-Nagel Nucleodur column (4.6 × 100 mm, 3 µm) at a flow rate of 1 $mL.min^{-1}$. The mobile phase was composed of 0.1% (v/v) TFA-H_2O (Solvent A) and 0.1% TFA-CH_3CN (Solvent B). Detection was performed at three different wavelengths (200, 214 and 254 nm) and the column temperature in the oven was maintained at 50°C. Semi-preparative purifications of oligoureas were performed on a Gilson GX-281 system using a Macherey-Nagel Nucleodure column (20 × 250 mm, 5 µm) at a flow rate of 20 $mL.min^{-1}$. The mobile phase composition was similar to the one used for analytical injections. Column effluent was monitored by UV detection at 200 and 214 nm. The purity of the compounds was determined to be ≥ 95. Oligomers were characterized by electrospray ionization low- and high-resolution (ESI, HRMS) obtained from the Mass Spectrometry facility at the European Institute of Chemistry and Biology (IECB, UMS3033),Pessac, France.
